# The strength of the antibody response to the nematode *Ascaris lumbricoides* inversely correlates with levels of B-Cell Activating Factor (BAFF)

**DOI:** 10.1186/1471-2172-15-22

**Published:** 2014-06-07

**Authors:** Adriana Bornacelly, Dilia Mercado, Nathalie Acevedo, Luis Caraballo

**Affiliations:** 1 Institute for Immunological Research, University of Cartagena, Cra 5, #7-77, 13-0015 Cartagena, Colombia; 2 Foundation for the Development of Medical and Biological Sciences (Fundemeb), 13-0001 Cartagena, Colombia

**Keywords:** *Ascaris*, Antibodies, Asthma, BAFF, BAFF-R, IgE, IgG, Immune response, Nematode, Parasite

## Abstract

**Background:**

B-Cell Activating Factor (BAFF) is a cytokine regulating antibody production. Polymorphisms in the gene encoding BAFF were associated with the antibody response to *Ascaris* but not to mite allergens. In the present study we evaluated the relationship between BAFF and specific antibodies against *Ascaris* and mites in 448 controls and 448 asthmatics. Soluble BAFF was measured by ELISA and *BAFF* mRNA by qPCR. Surface expression of BAFF and its receptor (BAFF-R) was analyzed by flow cytometry.

**Results:**

Individuals with specific IgE levels to *Ascaris* >75th percentile had lower levels of soluble BAFF; those with specific IgG levels to *Ascari*s >75th percentile had reduced *BAFF* mRNA. Total IgE and specific IgE to mites were not related to BAFF levels. There were no differences in soluble BAFF or mRNA levels between asthmatics and controls. There was an inverse relationship between the cell-surface expression of BAFF-R on CD19^+^ B cells and BAFF levels at the transcriptional and protein level.

**Conclusions:**

These findings suggest that differences in BAFF levels are related to the strength of the antibody response to *Ascaris*.

## Background

Infection by *Ascaris lumbricoides* (ascariasis) is one of the most prevalent helminthic diseases, affecting about 1.5 billion people worldwide. The immune response to this nematode has as hallmarks the induction of anti-*Ascaris* IgE/IgG antibodies and a strong Th2-driven inflammation [1]. Specific antibodies to *Ascaris* have been associated to parasite resistance [2-5] and both the immunoglobulin repertoire as well as the strength of the antibody responses are genetically regulated [6]. In mice, the production of IgE to the *Ascaris* resistance marker ABA-1 is HLA restricted [7]. In humans, two linkage scans identified a QTL (Quantitative Trait Loci) on chromosome 13q33.3 associated with the susceptibility to *A. lumbricoides*[8,9]. This region harbors the gene *TNFSF13B* (Tumor Necrosis Factor Ligand Superfamily, member 13b) encoding for the cytokine B-Cell Activating Factor, BAFF. The wild type allele of an intronic polymorphism in this gene (rs10508198) was found associated with higher IgG levels against *Ascaris* while the mutant allele was associated with less IgE to ABA-1 in asthmatics patients [10] suggesting a role of *TNFSF13B* in the antibody response to *Ascaris*.

BAFF (also known as BlyS, CD257), is a member of the tumor necrosis factor ligand superfamily of cytokines and is a major regulator of B cell activation, proliferation, differentiation, survival and immunoglobulin class-switching [11-13]. It is mainly expressed in innate immune cells such as neutrophils, macrophages and dendritic cells [14,15] but is also produced by non-hematopoietic cells [16,17]. This molecule may exist as cell surface-bound or soluble forms, the latter secreted after a furin cleavage [18,19]. Soluble BAFF interacts with three receptors: TACI (transmembrane activator and calcium-modulating cyclophilin ligand interactor), BCMA (B cell maturation antigen) and BAFF-R (BAFF receptor or BR3), the latter expressed on peripheral B cells [20,21]. *In vitro* experiments have demonstrated the critical role of BAFF on the production of IgA and IgG antibodies and the synergic effect with IL-4 on the transcription of IgE [22]. In these processes the BAFF-R also plays an important role [23,24].

Changes in BAFF levels are detectable in plasma during different immune related conditions [16]. In addition, there is evidence that upon nematode infection, resistant animals have increased levels of BAFF. In cattle naturally exposed to *Ostertagia*, *Cooperia* and *Nematodirus* the parasite-resistant animals had increased *BAFF* mRNA levels in the mesenteric lymph nodes [25]. High *BAFF* mRNA expression have been also detected in the spleen and liver of red grouse infected with *Trichostrongylus tenuis*[26]. Further studies in protozoan infections found that BAFF might be associated to variations in the strength of specific antibodies; for instance, administration of an antibody blocking BAFF signaling in mice infected with *Trypanosoma cruzi* induced a significant reduction of serum specific-IgM to the parasite [27]. Besides, there is evidence that *Plasmodium* parasites can modulate the BAFF pathway in the host, compromising protective antibody memory [28]. Although these investigations suggest that *TNFSF13B* may be a candidate gene underlying phenotypic variation in response to parasite infections, few have been done in humans. There are no studies evaluating the role of BAFF on the strength of *Ascaris* specific antibody production or total IgE in humans naturally exposed to this parasite. Since it has been observed that asthmatics have a higher antibody response to nematodes [29], these individuals are an interesting group for analyzing these traits. The aims of this study were (a) to investigate the relationship between BAFF and specific antibody levels to *Ascaris*, (b) to evaluate the relationship between BAFF and total IgE and specific IgE to non-parasitic allergens in asthmatic patients and non-asthmatic controls and (c) to evaluate the relationship between BAFF levels and the cell-surface expression of its receptor (BAFF-R) on CD19^+^ B cells.

## Results and discussion

### BAFF and the antibody response to *Ascaris*

**Figure 1:**
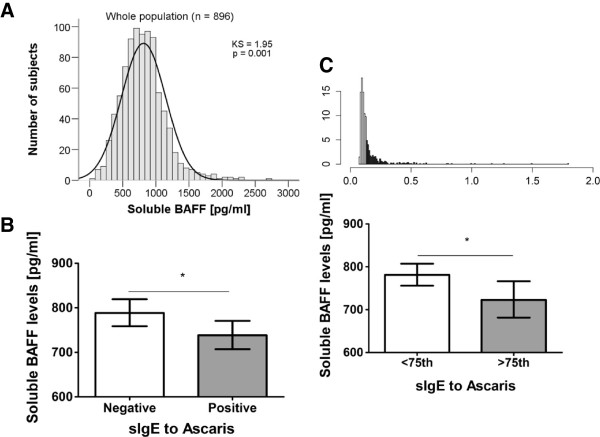
**BAFF levels according to the IgE antibody response to *Ascaris. *A**. Distribution of soluble BAFF levels in the population; KS: Kolmogorov-Smirnov **B**. Soluble BAFF levels in individuals with negative (n = 425) or positive (n = 392) IgE to *Ascaris***C**. Distribution of specific IgE to Ascaris; density (y-axis), optical density units (x-axis), shaded bars represent individuals with levels >75th percentile (upper panel). Soluble BAFF levels between individuals with specific IgE levels to *Ascaris* below (n = 583) and above (n = 234) 75th percentile (lower panel). All lines indicate geometric mean (95%CI), *p < 0.05.

The demographical characteristics and antibody responses of the studied population are presented in Table 1. In the entire dataset (n = 896), BAFF values in plasma were non-normally distributed with median 792.1 pg/ml (interquartile range 594.2–978.7 pg/ml) Figure 1A. There were no differences in soluble BAFF levels according to age, gender or disease status. IgE to *Ascaris* was higher in asthmatic patients while IgG to *Ascaris* was higher in controls and total serum IgE was higher in asthmatics (Table 1).

**Table 1:** Descriptive of the study population

**Variables**	**Asthmatic patients (n = 448)**	**Non-asthmatic controls (n = 448)**	**p-value**
Age, years (Mean ± SD)	34.4 ± 18.2	36.7 ± 18.6	0.06
Gender, female, n (%)	256 (57.1)	249 (55.6)	0.6
Soluble BAFF levels [pg/ml]^a^	768 (554.2–999.1)	804.3 (628.5–964.3)	0.16^b^
mRNA levels *BAFF1*^c^ (Mean ± SD)	3.21 ± 1.39	3.12 ± 1.14	0.6^d^
mRNA levels *BAFF2*^c^ (Mean ± SD)	2.79 ± 1.04	2.70 ± 0.94	0.5^d^
Total IgE, UI/ml^a,e^	699.7 (236.3–1065.8)	148.8 (58.3–408.1)	<0.001^b^
*Ig levels to parasite (OD)*^a,e^			
sIgE to *Ascaris*	0.117 (0.100–0.150)	0.105 (0.091–0.134)	<0.001^b^
sIgG to *Ascaris*	1.99 (1.60–2.34)	2.17 (1.73–2.69)	<0.001^b^
sIgE to ABA-1	0.125 (0.098–0.187)	0.119 (0.099–0.154)	0.18^b^
sIgG to ABA-1	1.49 (1.09–1.83)	1.70 (1.36–2.11)	<0.001^b^
*Ig levels to HDM (OD)*^a,e^			
IgE to *D. pteronyssinus*	0.191 (0.115–0.520)	0.099 (0.089–0.122)	<0.001^b^
IgE to *B. tropicalis*	0.231 (0.110–1.06)	0.098 (0.089–0.122)	<0.001^b^

SD: Standard deviation.

^a^Median (Inter Quartile Range).

^b^Mann-Whitney U test.

^c^Measured in PBMCs (n = 131, including 71 asthmatics and 60 controls).

^d^Independent samples t-test.

^e^Data available only for 369 asthmatics.

The relationship between levels of soluble BAFF and antibodies to *Ascaris* was first evaluated using bivariate correlations stratified by healthy and asthmatics. Because soluble BAFF and the specific IgE levels to *Ascaris* were non-normally distributed, the non-parametric Spearman test was used. We found a significant inverse correlation between soluble BAFF and specific IgE to *Ascaris* (r = -0.10, p = 0.03) in the group of healthy individuals (n = 448). A similar tendency was observed when comparing soluble BAFF levels between subjects with positive IgE sensitization to *Ascaris* (OD ≥ 0.113, n = 392) and those non-sensitized (n = 425): soluble BAFF was lower in *Ascaris* sensitized (median 786.3 pg/ml; IQR 595.1–973.6) than in non-sensitized (829.6 pg/ml; IQR 653.3–1013, Mann-Whitney U test, p = 0.03), Figure 1B.

Considering that parasite loads are over-dispersed within populations [30] and that individuals at the extreme of the distribution might carry biological traits influencing parasite susceptibility, we analyzed the relationship between soluble BAFF and the strength of the antibody response by stratifying the participants according their antibody levels, above 75th percentile (>75th) and below 75th percentile (<75th), Figure 1C (upper panel). We found that those with high specific IgE levels to the *Ascaris* extract (n = 234) had less concentrations of soluble BAFF in plasma (median 786.3 pg/ml; IQR 573.8–985) than those with IgE levels <75th percentile (n = 583, median 819.7 pg/ml; IQR 653.6–991.3; Mann-Whitney U test p = 0.04), Figure 1C. This association remained significant in the linear regression model using the square-root transformed soluble BAFF levels, and after adjustment by age, gender and disease status (p = 0.028). To explore if this relationship was reflected at the gene expression level, we analyzed a sub-group of subjects (n = 131) with mRNA data in peripheral blood mononuclear cells (PBMCs), but we found no association between the relative expression of *BAFF* mRNA and specific IgE levels to *Ascaris* (data not shown).

**Figure 2:**
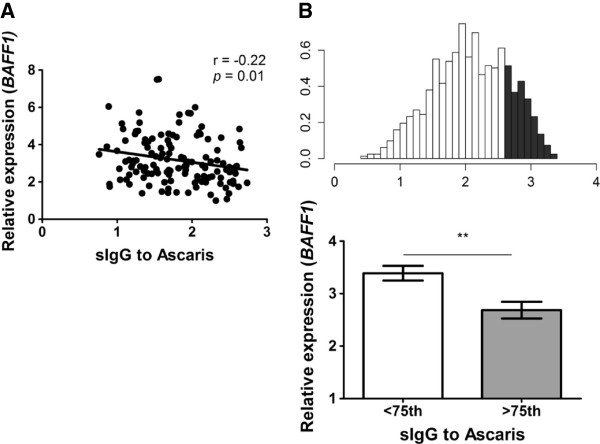
***BAFF *mRNA levels according to the specific IgG to *Ascaris. *A**. Correlation between the mRNA expression for *BAFF1* and the levels of specific IgG to *Ascaris*. The numbers indicate the Pearson correlation coefficient (r) and the p-value (two-tailed); each dot represents an individual **B**. Distribution of specific IgG levels to Ascaris; density (y-axis), optical density units (x-axis), shaded bars represent individuals with IgG levels >75th percentile (upper panel); *BAFF1* mRNA levels between individuals with IgG to *Ascaris* below (n = 91) or above (n = 40) 75th percentile (lower panel), **p < 0.005.

Besides, there was no significant correlation between soluble BAFF levels and IgG levels to Ascaris using non-parametric tests. Since IgG levels were normally distributed, we tested a more powerful parametric test (Pearson’s r) using square-root-transformed soluble BAFF levels, with similar results. Interestingly, there was a significant inverse correlation between *BAFF* mRNA levels and IgG to *Ascaris* (r = - 0.22, p = 0.01), Figure 2A. This relationship was also observed when the comparison was done according to the strength of the antibody response: individuals with specific IgG levels to *Ascaris* >75th percentile had lower *BAFF1* mRNA levels (n = 40, mean 2.68 ± 1.0 SD) than those < 75th percentile (n = 91, mean 3.38 ± 1.3 SD, p = 0.003), Figure 2B.

The relationship between BAFF and serum antibody levels has been extensively analyzed in the context of autoimmune diseases, where increased levels of soluble BAFF have been associated with increased titers of auto-antibodies of the IgG isotype [11,31-34]. However, the effects of BAFF on specific antibody levels may differ depending on the context, for instance, inverse relationships between *BAFF* mRNA levels and the risk of developing donor-specific antibodies have been observed after transplantation [35]. To our knowledge this is the first study suggesting a relationship between circulating BAFF levels and the human antibody response to a nematode. In contrast to what is generally observed in autoimmune diseases and some parasitic infections, we found an inverse association between soluble BAFF and the strength of the antibody response to *Ascaris*. The fact that the same tendency was observed both at protein and mRNA level is highly suggestive that it represents a biological phenomenon. The mechanisms underlying this relationship are unknown.

The mean difference in soluble BAFF levels between subjects with high specific IgE to *Ascaris* and the rest of the population was 52 pg/ml. The biological significance of this finding need to be further investigated but there are several studies suggesting that it could be relevant. It has been shown that most of the systemic BAFF is bound to receptors on B cell surfaces and that BAFF binding capacity on follicular B cells is nearly saturated under steady-state conditions in vivo [36]. In this scenery a small change in circulating BAFF concentrations may affect the BAFF balance in lymphoid tissues and impair the survival of high-affinity B cells clones in the germinal center. In addition, it is known that for some cytokines the biological impact is in the order of few pg/ml; for example, concentrations of IFNγ between healthy and malaria infected children differ in 8.1 pg/ml, and this small change correlated with soluble BAFF levels and concentration of antibodies [37]. Moreover, differences in cytokine levels upon stimulation of PBMCs with phytohemagglutinin or *Ascaris* were small but the impact on antibody production was remarkable [38]. Differences in levels of pro-inflammatory cytokines between healthy individuals and patients with autoimmune conditions have been also described in this range [39].

Previous studies showed that *TNFSF13B* gene is a QTL for *Ascaris* susceptibility [8,9], harboring genetic variants associated with IgE response to *Ascaris* and worm burden [10,40]. Now we have data suggesting that, in addition to genetic evidence, there is a relationship between the antibody responses to *Ascaris* and BAFF at the protein and mRNA level; although a causal relationship between them has yet to be demonstrated. Our findings seem to be more related to genetic mechanisms because first, there is previous evidence suggesting that polymorphisms around *TNFSF13B* may influence the antibody responses and second, we also found an association between the IgG responses and the level of mRNA expression. It is also pertinent to consider that *A. lumbricoides* can modify BAFF levels during infection, as has been reported for malaria parasite in acutely infected children [37]. However, in our study it is not possible to assess a direct effect of the parasite on BAFF production because it was designed to evaluate the antibody responses to *Ascaris* and not the acute infection. Besides, we should keep in mind that low BAFF levels and high antibody responses might be independent consequences of the *Ascaris* infection without any causal relationship. Indeed, IL-4 has shown to down-regulate the expression of BAFF *in vitro*[41]. It is known that *Ascaris* infections of low intensity are associated with a Th2 immune response rich in IL-4 and in this population this could be an additional factor introducing variability in BAFF levels.

### Soluble BAFF, total IgE, specific IgE and asthma

**Figure 3:**
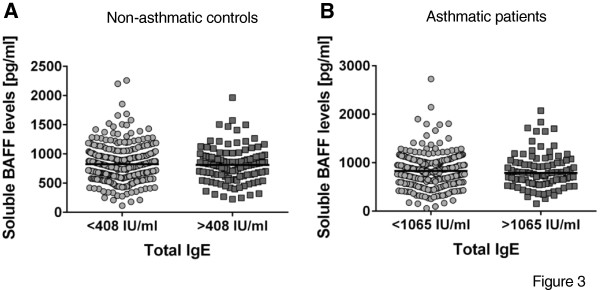
**Soluble BAFF levels according to total IgE levels. A**. Soluble BAFF levels in non-asthmatic controls according to total IgE below (n = 335) or above (n = 113) 75th percentile **B**. Soluble BAFF levels in asthmatics according to total IgE below (n = 277) or above (n = 92) 75th percentile.

The relationship between BAFF and total plasma IgE is still controversial [42-45]. In this study we found no association between soluble BAFF and total IgE when analyzed as continuous variables. Total IgE values were transformed to a categorical variable based on the 75th percentile in non-asthmatic controls and asthmatic patients as a cut-off to define high total IgE levels but there were no significant differences (Figure 3). In addition, BAFF mRNA levels were not associated with total IgE. Our findings are in agreement with previous studies showing no relationship between BAFF levels and total IgE [43]. Regarding specific IgE to non-parasite/environmental allergens, we found that BAFF levels were not related to specific IgE to *D. pteronyssinus* and *B. tropicalis*, the main sensitizers in this population. There are contradictory published results around this point: it has been reported a significant increase in BAFF levels after allergen exposure in the bronchoalveolar lavage fluid of allergic patients [46] but no relationship was found between soluble BAFF and specific IgE reactivity to fungal allergens [42]. Our results support previous findings detecting association between BAFF variants and specific IgE to *Ascaris* but not to mites [10]. Some studies have suggested a relationship between BAFF and asthma in humans [44,47]. However, in agreement with Lei *et al.*[48], we found no differences in BAFF between asthmatic patients and controls neither at protein nor at the mRNA levels (Table 1). It is worth mentioning that our results refer to circulating form of the cytokine and its expression in peripheral blood leukocytes. Although several covariates might influence BAFF synthesis, the levels we found in healthy controls were comparable to those observed in other populations [49-51].

### *BAFF* mRNA levels inversely correlate with the cell surface expression of BAFF receptor (BAFF-R) on B cells

**Figure 4:**
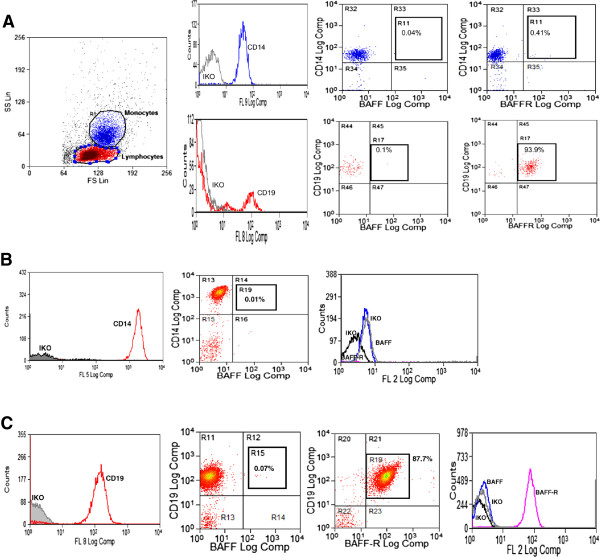
**Cell surface expression of BAFF and BAFF-R in PBMC, monocytes and B cells. A**. Cell surface expression of BAFF and BAFF-R on gated monocytes (CD14^+^) and B cells (CD19^+^). A representative example out of 113 PBMCs samples tested. IKO: isotype control **B**. Cell surface expression of BAFF and BAFF-R in purified monocytes **C**. Cell surface expression of BAFF and BAFF-R in purified B cells.

**Figure 5:**
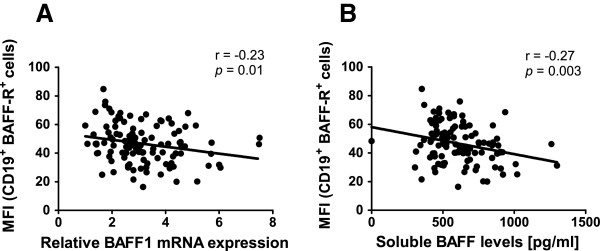
**Relationship between cell surface expression of BAFF-R and BAFF levels. A**. Correlation between the median fluorescence intensity (MFI) of BAFF-R on gated CD19^+^ B cells and the mRNA levels for *BAFF1* in PBMCs **B**. Correlation between the MFI of BAFF-R on gated CD19^+^ B cells and soluble BAFF levels in plasma. The numbers indicate the Pearson correlation coefficient (r) and the p-value (two-tailed). Each dot represents an individual.

Since there were previous reports showing an inverse relationship between BAFF and BAFF receptor [50,52,53], the expression of membrane-bound BAFF and BAFF-R was evaluated on PBMCs (n = 113). We did not detect cell surface expression of BAFF in gated CD14^+^ cells (monocytes), lymphocytes or gated CD19^+^ cells (B cells); and, as previously described [54], the BAFF-R was highly expressed in peripheral B cells but not in monocytes (Figure 4A). Similar findings were obtained when cell-surface expression of BAFF and BAFF-R were analyzed in sorted monocytes (Figure 4B) and B cells (Figure 4C) from six non-asthmatic controls. As expected [54] we found that plasmablasts (CD27^high^, CD38^high^), switched-memory B cells, mature naïve B cells and transitional B cells expressed BAFF receptor (Additional file 1). The relationship between the cell-surface expression of BAFF-receptor (BAFF-R) on CD19^+^ B cells (median fluorescence intensity, MFI) with the soluble BAFF levels and the BAFF mRNA levels in PBMCs was evaluated in the subgroup of 113 individuals with flow cytometry data. These observations had a normal distribution in this dataset and therefore parametric tests (Pearson’s correlation) were used (Additional file 2). We found a significant inverse correlation between *BAFF1* mRNA and the cell surface expression of BAFF-R in CD19^+^ B cells (r = - 0.23, p = 0.01), Figure 5A. This finding was independent of age, gender, disease status and the proportion of monocytes in the sample as tested in a linear regression model. However, no correlation was found with *BAFF2* mRNA levels (r = - 0.13, p = 0.1). Regarding protein levels, we found an inverse correlation between soluble BAFF levels and the cell surface expression of BAFF-R in CD19^+^ B cells (r = -0.27, p = 0.003) Figure 5B. These observations were significant after adjustment by age, gender, disease status and the proportion of monocytes in the sample. The inverse relation between soluble BAFF levels and the cell surface expression of BAFF-R has been previously reported in mice and patients with deficiency of BAFF-R [50], patients with systemic lupus erythematous and Sjogren’s syndrome [52] hyper-IgE syndrome [53] and acute malaria [37]. In this model, the 8.2% of the variance in BAFF-R expression can be explained by the soluble BAFF levels. In the context of immune responses to *Ascaris*, it remains to be elucidated how this BAFF/BAFF-R axis is related to the synthesis of specific antibody levels.

### The relationship between circulating soluble BAFF levels and *BAFF* mRNA levels in blood mononuclear cells

**Figure 6:**
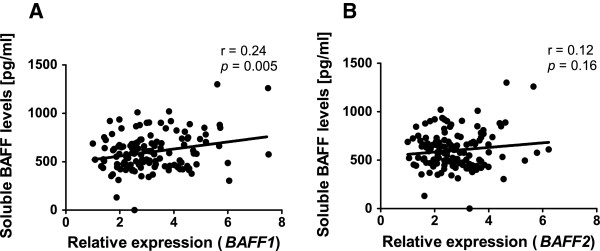
**The relationship between soluble BAFF levels and *BAFF *mRNA levels in blood mononuclear cells. A**. Correlation between soluble BAFF levels and *BAFF1* mRNA expression **B**. Correlation between soluble BAFF levels and *BAFF2* mRNA expression. The numbers indicate the Pearson correlation coefficient (r) and the p-value (two-tailed). Each dot represents an individual.

Another question to be addressed was the relationship between soluble BAFF and BAFF mRNA levels, and whether those can serve as proxies for mechanisms contributing to the variation in specific antibody levels. Immune responses to antigens including helminths take place in lymphoid tissues and in infiltrated organs. There, BAFF is produced by many cells including lymphoid tissue stroma cells and resident monocytes [55,56]. Levels of circulating soluble BAFF has shown to correlate with local BAFF activity at the germinal center [36]. In this study we found a significant positive correlation (r = 0.68, p < 0.001), between the mRNA levels for *BAFF1* (exon 3-4) and *BAFF2* (exon 4-6), however, soluble BAFF levels were only significantly correlated to *BAFF1* mRNA levels (Figure 6A) and not to *BAFF2* mRNA (Figure 6B). From the transcripts to soluble BAFF there are several steps: as a type II transmembrane protein of 285 amino acids (aa) in the plasma membrane, BAFF is cleaved by furine proteases in its N-terminal end (TNF-homology domain) and released in the circulation. There it forms homotrimers of 152 aa amenable to be detected by ELISA; homotrimers can also form other assemblies of less abundance (i.e. BAFF 60 mer) [16,57]. There is another isoform expressed at low levels and called ∆BAFF (266 aa), that can form heteromultimers with BAFF (285 aa) and negatively regulate the secretion of the latter [58]. The relation between soluble BAFF levels and *BAFF1* mRNA levels could be explained because this transcript contains exon 3, which is informative for both BAFF and ∆BAFF. More studies are needed to elucidate if differential expression of BAFF isoforms contribute to the strength of specific antibody levels.

## Conclusions

Our findings show that soluble BAFF levels are lower in subjects with high specific IgE to *Ascaris*, suggesting that this cytokine plays a role in the strength of the antibody responses to this nematode and supporting previous molecular genetics studies. Interestingly, soluble BAFF levels are not related with total IgE or IgE sensitization to house dust mites, also in agreement with our previous findings. In addition, we found no differences in BAFF levels between asthmatic patients and controls. As mRNA, BAFF was expressed in mononuclear cells but, as a protein it was not detected in cell-surface including monocytes and B cells. The potential mechanistic link between lower levels of BAFF and the strength of the specific antibodies to *Ascaris* might be related with the inverse relationship between BAFF levels and the BAFF-R expression on B cells, a receptor that was present at the cell surface in all developmental stages of peripheral B cells including switched memory and plasmablasts. The role of BAFF on the susceptibility to *Ascaris* infection and its clinical implications deserve further confirmatory and mechanistic studies in larger cohorts.

## Methods

### Study design and population

This is a case-control study performed in Cartagena, Colombia. Eight hundred ninety six subjects including 448 asthmatics and 448 non-related healthy volunteers were selected from a well-characterized dataset [10] and matched by age and gender (Table 1). The matching process was done randomly, without knowledge of anti-*Ascaris* IgE or IgG levels. All participants live in an urban, non-industrialized setting, having access to water and electricity and belonging to the lower three (of six) socio-economic strata in the city, where most people are naturally exposed to *A. lumbricoides*. Measurements of soluble BAFF (n = 896) were done in non-previously thawed plasma samples from the repository in which anti-*Ascaris* antibody determinations were done previously [10]. A random subgroup of these subjects (n = 131, 71 asthmatics and 60 controls) were visited by a physician of the research staff who obtained 20 mL blood samples for gene expression, flow cytometry analyses, soluble BAFF, total IgE and specific antibodies to *Ascaris* and ABA-1. Asthma diagnosis was confirmed according GINA guidelines, including spirometry. Allergic sensitization was defined as a positive IgE result to the mites *Dermatophagoides pteronyssinus* and/or *Blomia tropicalis*. The study was approved by the Ethics Committee of the University of Cartagena. A full verbal explanation of the investigation was given and written informed consent was obtained from all participants.

### Antibody measurements

Total IgE was determined by duplicate using an enzyme-linked immunosorbent assay (ELISA) kit (RIDASCREEN; R-Biopharm, Darmstadt, Germany) according to the manufacturer’s instructions. Specific IgE and IgG to *Ascaris* extract and ABA-1 as well as specific IgE to mite extracts were detected by ELISA as described previously [10,59]. ABA-1 is a nematode specific allergen from *Ascaris* (Asc s 1) that is useful for avoiding cross reactivity with mite allergens [59]. Also, antibody responses to ABA-1 have been associated with resistance to ascariasis [3,5]. The responses to *Ascaris* and ABA-1 were analyzed as dichotomous variables, using two cut-off points: the first was the optical density (OD) value of 0.113 (mean OD of six negative, non-allergic, non-parasitized controls + 3 standard deviations) which defined sensitized (positive) and non-sensitized (negative) individuals. The second was the 75th percentile of antibody levels in non-asthmatics individuals (i.e., 0.134 OD units for IgE to *Ascaris*; 0.154 OD units for IgE to ABA-1; 2.68 OD units for IgG to *Ascaris* and 2.11 OD units for IgG to ABA-1). To analyze the strength of total IgE levels the 75th percentile was used as cut-off to define high total IgE phenotype. This percentile was calculated separately in healthy controls (>75th = 408.1 IU/mL) and asthmatics (>75th = 1065.8 IU/mL).

### Quantification of BAFF levels in plasma

Soluble BAFF in plasma was measured using a quantitative sandwich enzyme immunoassay according to the manufacturer’s instructions (Quantikine Human BAFF/BLyS kit, R&D Systems Cat. SBLYS0). A standard curve with four parameter logistic (4-PL) curve-fit was used to extrapolate the sample concentration value. Intra assay variation coefficients were lower than 10%. The quantification range of the assay was between 62.5 and 4000 pg/ml.

### Isolation of peripheral blood mononuclear cells (PBMCs)

Peripheral blood was diluted 1:2 in 1× PBS and separated by density gradient using Ficoll Histopaque (Sigma Aldrich, Catalog 1077-1). Tubes were centrifuged during 30 minutes at 400 *g* at room temperature and without brake. The layer containing the PBMCs was transferred into a new tube, washed twice with 50 mL of 1× PBS and resuspended in 1 mL of 1× PBS. A 10μl aliquot was stained with 0.4% Trypan Blue and counted in a hemocytometer. For gene expression analysis, 1 × 10^7^ cells were placed in a new tube, centrifuged at 300 *g*, 4°C during 5 minutes. The supernatant was discarded and the cell pellet homogenized in 750 μl of Trizol® (Life Technologies) and stored at -70°C until use. The remaining cells were used for flow cytometry analysis (see below).

### RNA extraction

Cell homogenates were thawed during 15 minutes at room temperature (protected from light) and 150 μl of chloroform were added to each tube, vigorously mixed by hand and centrifuged at 12 000 *g* during 15 minutes at 4°C. The aqueous upper phase was transferred to a fresh tube, diluted in 400 μl of 70% ethanol and pipetted into a spin column (Trizol® Plus RNA purification kit, Life Technologies, Catalog: 12183555). Each sample was centrifuged at 12000 *g* during 15 seconds at room temperature, the flow-through was discarded and the column containing the RNA washed three times. The spin column was centrifuged at 12 000 *g* during 1 minute and 50 μl of RNAse free water were added for RNA elution (12 000 *g* during 2 minutes at room temperature). The RNA yield was between 8.1 μg and 25 μg. The A260/A280 ratio ranged from 1.92 to 2.03 and for the ratio A260/A230 from 0.71 to 2.36. Integrity of RNA was also verified by electrophoresis as two intact bands of 28S and 18S ribosomal RNA.

### cDNA synthesis

cDNA was synthesized from 1 μg of total RNA using the Superscript III first strand super mix kit (Invitrogen, Catalog 11752050) following manufacturer’s instructions. RT-PCR reactions included retrotranscriptase, reaction mix (oligodT 22.5 μM, random hexamers 2.5 ng/μl, 10 mM MgCl_2_ and dNTPs), total RNA and RNAse-free water (Ambion Cat. AM9937). The synthesized cDNA was diluted with 80 μl of water and stored at -20°C until amplification. cDNA was genomic-DNA free, confirmed by an amplification reaction using a non retrotranscriptase enzyme control (RT-minus control).

### Quantitative PCR

BAFF mRNA was detected by quantitative PCR (qPCR) using Taqman gene expression assays on a 7300 Real-Time Polymerase Chain Reaction (PCR) system (Applied Biosystems, Foster City, CA, USA). To detect differential splicing, the mRNA was measured in two separated assays: assay 1 targeting the exons 3 and 4 (Cat. Hs00198106, 84 bp amplicon) and assay 2 targeting the boundaries of exons 4–5 and 5–6 (Cat. Hs00902574, 73 bp amplicon). The two isoforms were named *BAFF1* and *BAFF2* (Additional file 3). Expression of β2 micro-globulin was used as endogenous control (Cat. Hs00984230, 81bp amplicon). Each sample was tested by duplicate and the average C_T_ value exported from the SDS Software (Applied Biosystems). The C_T_ value of the β2M gene (Mean C_T_ 18.8 ± 0.40 SD) was subtracted from the C_T_ values of the target genes (Mean C_T_ 25.7 ± 0.60 SD for *BAFF1* and Mean C_T_ 27.1 ± 0.67 SD for *BAFF2*). The normalized value was expressed as the delta C_t_ (ΔCt = Ct_BAFF_ – Ct_B2M_). The highest ΔC_T_ value among all samples was subtracted from each sample and the resulting number expressed as the *delta-delt*a C_T_. Relative expression levels were calculated as 2^^-(ΔΔCT)^[60]. The probes for BAFF were FAM-labeled and for β2 micro-globulin were VIC-labeled, all assays included NFQ (non- fluorescent quencher).

### Flow cytometry on PBMCs

Cell surface expression of BAFF and BAFF-R was analyzed on PBMCs by flow cytometry (n = 113). Cells were resuspended in MACS buffer (0.5% BSA in PBS) at a final concentration of 10^5^ cells/100 μl per tube. Fcγ receptors were blocked with 1 μl of normal mouse serum (e-Bioscience, Catalog 24-5544) during 10 minutes at 4°C. A panel of fluorochrome-conjugated monoclonal antibodies (APCeFluor α-human CD14, Catalog: 47-0149; APC α-human CD19, Catalog 17-0199; PE α-human BAFF/BLyS, Catalog 12-9017 and FITC α-human BR3/BAFF-R, Catalog 11-9117, all from e-Bioscence) were added to the cells and incubated 30 minutes at 4°C protected from light. Each assay included the unstained sample and a panel of appropriate isotype controls to set the gates of positive and negative populations. After staining, cells were washed twice with PBS and resuspended in 500 μl of PBS. Data on 20000 events per sample were acquired using a Dako Cytomation Cytometer (Beckman Coulter, Inc. CA, USA) and analyzed by Summit 4.3 (Beckman Coulter, Inc. CA, USA). Electronic gating was used to evaluate the expression of BAFF and BAFF-R in CD14^+^ cells and CD19^+^ B cells.

### Flow cytometry in purified monocytes and B cells

Peripheral blood monocytes and B cells were purified by magnetic associated cell sorting (MACS) using negative selection protocols (Monocyte Isolation kit catalog 130-091-153 and B cells isolation kit catalog 130-091-151, Miltenyi Biotec). Briefly, 30 × 10^6^ PBMCs were resuspended in MACS buffer containing 0.5% BSA - PBS pH 7.2. Then, biotin-conjugated antibody cocktails were added and the mixture incubated by 10 minutes at 4°C. Non target cells were magnetically labeled with a cocktail of monoclonal antibodies targeting CD2, CD14, CD16, CD36, CD43 and CD235a for the B cell separation protocol and CD3, CD7, CD16, CD19, CD56, CD123 and CD235a for the monocyte separation protocol (Miltenyi Biotec). Anti-biotin microbeads were added and the cells incubated by 15 minutes at 4°C. After washing, cells were resuspended in MACS buffer at a final volume of 500 μl and passed through the column. The flow-through was collected and centrifuged at 300 *g* during 5 minutes at 4°C. The supernatant was discarded, and the cells reconstituted in 500 μl of MACS buffer and counted. Purified cells were stained with specific antibodies to detect BAFF and BAFF-R in different subpopulations (Additional file 4). The purity of monocytes and B cells was 91% and 90% respectively.

### Statistics

Statistical analyses were performed using the statistical package for the social sciences software (SPSS version 17 for Windows; SPSS Inc., Chicago, IL, USA) and GraphPad Prism software v.5. Bivariate correlations were used to analyze the relationship between soluble BAFF and the antibody levels as continuous variables; since soluble BAFF was non-normally distributed, the non-parametric Spearman test was used. IgG levels were normally distributed therefore soluble BAFF levels were transformed to their square root to achieve normality and the Pearson correlation test was applied for this isotype. The comparisons of soluble BAFF between the groups of antibody responders (<75th and >75th) were done using Mann-Whitney *U* test (p-values presented after 10000 permutations). Linear regression was then used to model the effect of antibody grouping on transformed soluble BAFF levels adjusting by age, gender and disease status (asthma yes/no). Since *BAFF* mRNA values in PBMCs were normally distributed (Additional file 2), the comparison between groups of antibody responders (<75th and >75th) were done by independent samples *t* test and further verified by adjusted linear regression models. MFI of BAFF-R was also normally distributed and its relationship with soluble BAFF and transcript levels were explored using Pearson correlation test. Since BAFF transcripts were measured in unfractionated mononuclear cells, the percentage of monocytes per sample was included as covariate in adjusted linear models. The relationship between soluble BAFF levels and the mRNA levels of the transcripts *BAFF1* and *BAFF2* was analyzed using Pearson correlation test. A p value ≤0.05 (two-tailed) was considered statistically significant.

## Competing interests

The authors declare that they have no competing interests.

## Authors’ contributions

AB, NA performed experiments and statistical analyses; DM performed antibody measurements; NA participated in the study design and supervised data analysis. LC conceived the investigation and supervised the general aspects of the work and data analyses. AB, NA, LC wrote the manuscript. All authors read and approved the final version of the manuscript.

## Supplementary Material

Additional file 1 Cell surface expression of BAFF-R in subpopulations of purified B cells. Click here for file

Additional file 2 Distribution of the median intensity levels of BAFF-R in B cells, soluble BAFF levels and mRNA levels in PBMCs for the subgroup of 113 individuals. Click here for file

Additional file 3 Schematic representation of the gene encoding BAFF and the exons covered by the gene expression assays. Click here for file

Additional file 4 Antibodies used for flow cytometry in PBMCs, monocytes and B cells. Click here for file
