# Supplementary material for: The strength of the antibody response to the nematode Ascaris lumbricoides inversely correlates with levels of B-Cell Activating Factor (BAFF)
Source: BMC Immunol. 2014 Jun 7;15:22. doi: 10.1186/1471-2172-15-22 (PMC4067067; doi:10.1186/1471-2172-15-22)
Supplement: Additional file 1 — Cell surface expression of BAFF-R in subpopulations of purified B cells. [file 1471-2172-15-22-S1.docx]

Additional file 1: Figure S1

Cell surface expression of BAFF-R in sub-populations of purified B cells


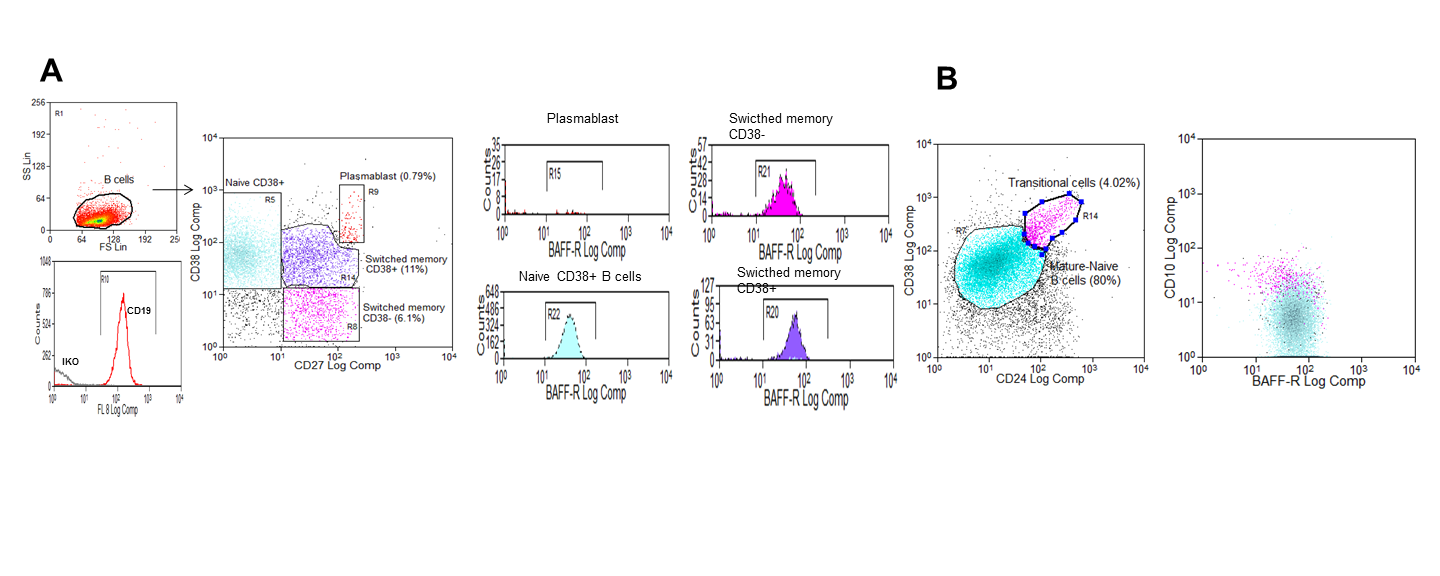


**A.** Cell surface expression of BAFF-R in plasmablasts, switched memory CD38^+^ and CD38^-^ and naïve B cells **B.** Cell surface expression of BAFF-R in transitional and mature naïve B cells.
