# Supplementary material for: The strength of the antibody response to the nematode Ascaris lumbricoides inversely correlates with levels of B-Cell Activating Factor (BAFF)
Source: BMC Immunol. 2014 Jun 7;15:22. doi: 10.1186/1471-2172-15-22 (PMC4067067; doi:10.1186/1471-2172-15-22)
Supplement: Additional file 2 — Distribution of the median intensity levels of BAFF-R in B cells, soluble BAFF levels and mRNA levels in PBMCs for the subgroup of 113 individuals. [file 1471-2172-15-22-S2.docx]

**Additional file 2: Figure S2**


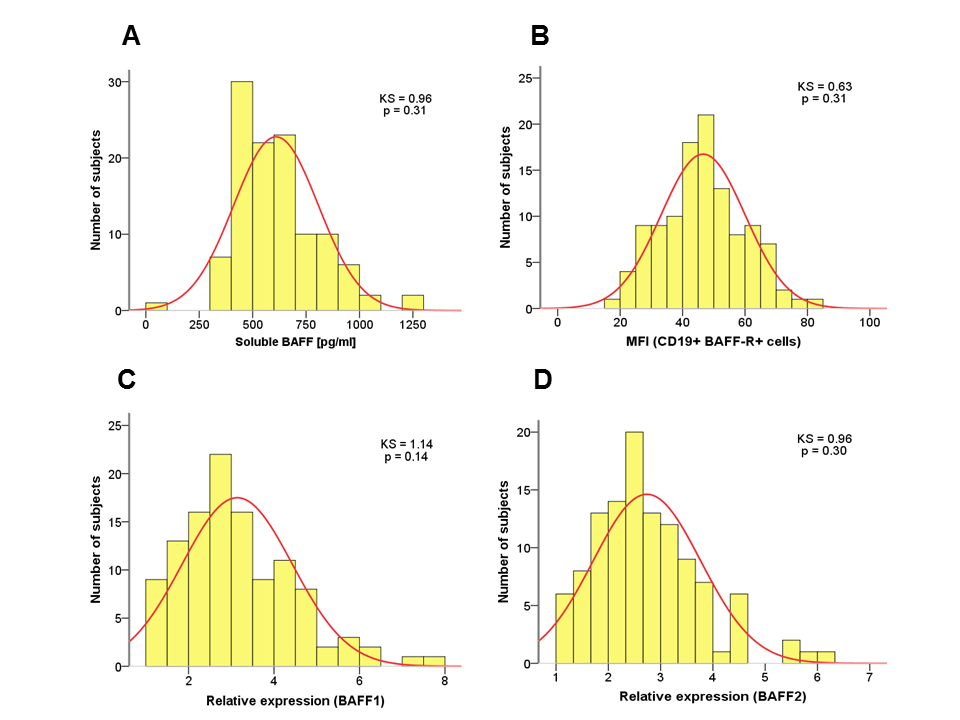


Distribution of variables in a subgroup of individuals with mRNA and flow cytometry data (n = 113) **A.** Soluble BAFF levels in plasma **B.** Median fluorescence intensity of BAFF-R on gated CD19^+^ B cells **C** *BAFF1* mRNA expression, and **D** *BAFF2* mRNA expression.
