# Supplementary material for: The strength of the antibody response to the nematode Ascaris lumbricoides inversely correlates with levels of B-Cell Activating Factor (BAFF)
Source: BMC Immunol. 2014 Jun 7;15:22. doi: 10.1186/1471-2172-15-22 (PMC4067067; doi:10.1186/1471-2172-15-22)
Supplement: Additional file 3 — Schematic representation of the gene encoding BAFF and the exons covered by the gene expression assays. [file 1471-2172-15-22-S3.docx]

**Additional file 3: Figure S3**

Schematic representation of the gene encoding BAFF and the exons covered by the gene expression assays


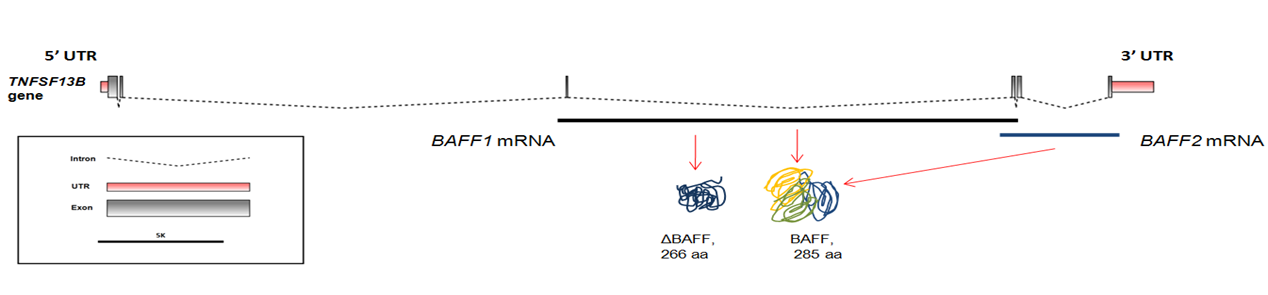


To take into account differential splicing, the mRNA levels for BAFF were measured by two separated assays: Assay 1 (*BAFF1*) targeted the exons 3 and 4 (Cat. Hs00198106, 84 bp amplicon) and Assay 2 (*BAFF2*) targeted the boundaries of exons 4-5 and 5-6 (Cat. Hs00902574, 73 bp amplicon). Both transcripts were correlated (r = 0.68, p < 0.001).
