# Supplementary material for: The strength of the antibody response to the nematode Ascaris lumbricoides inversely correlates with levels of B-Cell Activating Factor (BAFF)
Source: BMC Immunol. 2014 Jun 7;15:22. doi: 10.1186/1471-2172-15-22 (PMC4067067; doi:10.1186/1471-2172-15-22)
Supplement: Additional file 4 — Antibodies used for flow cytometry in PBMCs, monocytes and B cells. [file 1471-2172-15-22-S4.docx]

**Additional file 4: Table S1**

Antibodies used for flow cytometry in PBMCs, monocytes and B cells

| Specificity | Isotype | Catalog number | Clone | Manufacturer |
| --- | --- | --- | --- | --- |
| APC-F780 anti-human CD14 | IgG1k | 47-0149 | 61D3 | eBioscence |
| PECy7 anti-human CD14 | IgG2a | A22331 | RMO52 | Beckman Coulter |
| APC anti-human CD19 | IgG1k | 17-0199 | HIB19 | eBioscence |
| FITC anti-human BAFF | IgG1k | 11-9017 | 1D6 | eBioscence |
| PE anti-human BAFF | IgG1k | 12-9017 | 1D6 | eBioscence |
| FITC anti-human BAFF-R | IgG2a k | 11-9117 | 8A7 | eBioscence |
| PE anti-human BAFF-R | IgG2a k | 12-9117 | 8A7 | eBioscence |
| FITC anti-human CD24 | IgG2a k | 555427 | ML5 | BD |
| PE-Cy7 anti-human CD38 | IgG1k | 25-0389 | HIT2 | eBioscence |
| PE-Cy5 anti-human CD10 | IgG2b | 15-0106 | eBioCB-CALLA | eBioscence |
| APC anti-human CD27 | IgG1k | 17-0279 | O323 | eBioscence |
| PE-Cy7 anti-human CD27 | IgG1k | 25-0279 | 0323 | eBioscence |
| FITC anti-human IgG | IgG1 | MCA647F | MK1A6 | AbD serotec |
